# A Case Report of Pleomorphic Adenoma of the Lower Lip: An Uncommon Location and a Diagnostic Pitfall

**DOI:** 10.7759/cureus.116977

**Published:** 2026-09-26

**Authors:** Evangelos Parcharidis, Konstantinos Paraskevopoulos, Dimitrios Minotakis, Konstantinos Poulopoulos, Athanasios Poulopoulos

**Affiliations:** 1 Oral Medicine and Oral Pathology, School of Dentistry, Aristotle University of Thessaloniki, Thessaloniki, GRC; 2 Oral and Maxillofacial Surgery, Papanikolaou Hospital, Aristotle University of Thessaloniki, Thessaloniki, GRC; 3 Pathology and Laboratory Medicine, Genekor, Thessaloniki, GRC; 4 Oral Medicine and Maxillofacial Pathology, School of Dentistry, Aristotle University of Thessaloniki, Thessaloniki, GRC

**Keywords:** lower lip, minor salivary glands, oral medicine, pleomorphic adenoma, salivary gland tumors, submucosal lesion

## Abstract

Pleomorphic adenoma is the most common benign tumor of the salivary glands. Lower lip involvement is uncommon, and its nonspecific clinical appearance may complicate the diagnosis. We report a case of a man in his 30s who presented with a painless lower lip swelling that had gradually increased in size over several months. The lesion had initially been considered a sebaceous cyst and had been treated unsuccessfully with a topical corticosteroid. Clinical examination revealed an approximately 1-cm, firm, mobile, well-defined submucosal nodule covered by normal-appearing mucosa. Given the lesion's persistence and uncertainty regarding the clinical diagnosis, it was excised under local anesthesia and submitted for histopathological examination, which confirmed a pleomorphic adenoma with epithelial and myoepithelial components within a chondromyxoid stromal background. The postoperative course was uneventful, with no clinical evidence of recurrence during the six-month follow-up period; however, continued long-term clinical surveillance was planned. This case highlights the importance of including minor salivary gland tumors in the differential diagnosis of persistent lower lip swellings, even when they appear clinically benign, and underscores the key role of histopathological examination in establishing the diagnosis.

## Introduction

Pleomorphic adenoma is the most common benign salivary gland neoplasm [1]. Most cases arise in the parotid gland (around 80%), while a smaller proportion arises from the minor salivary glands [1]. The palate is the most frequently affected site, whereas lip involvement is relatively uncommon. Clinically, these tumors typically present as slow-growing, painless, well-defined masses [1]. Complete surgical excision with an adequate margin of surrounding normal tissue remains the treatment of choice [1]. Despite its benign nature, recurrence may occur, particularly after incomplete excision or capsular disruption during surgery [2]. Long-standing or recurrent lesions may also rarely undergo malignant transformation into carcinoma ex pleomorphic adenoma [3].

The uncommon occurrence of pleomorphic adenoma in the lower lip, combined with its nonspecific clinical presentation, may lead to diagnostic uncertainty. We present a case of pleomorphic adenoma of the lower lip that the patient's general dentist initially considered a sebaceous cyst before referral for specialist evaluation, highlighting the importance of including minor salivary gland tumors in the differential diagnosis of persistent submucosal lip lesions.

## Case presentation

A Greek man in his 30s was referred for specialist evaluation of a lower lip swelling that, according to the patient, had been present and gradually enlarging for several months, although the exact duration could not be established. The lesion was located on the mucosal aspect of the lower lip and was asymptomatic, with no history of pain, bleeding, or ulceration. It had previously been evaluated by his general dentist and was clinically considered a sebaceous cyst. The patient had therefore been treated with triamcinolone acetonide 0.1% oromucosal paste three times daily for seven days, without improvement in lesion size. His medical history was otherwise unremarkable, and he was not taking any regular systemic medication. No relevant family history of salivary gland disease was identified. There was no history of trauma to the lip, habitual lip biting, or similar lesions in the past. On clinical examination, a solitary nodular swelling was noted on the right mucosal aspect of the lower lip, opposite the lower right incisors (Figure 1).

**Figure 1:**
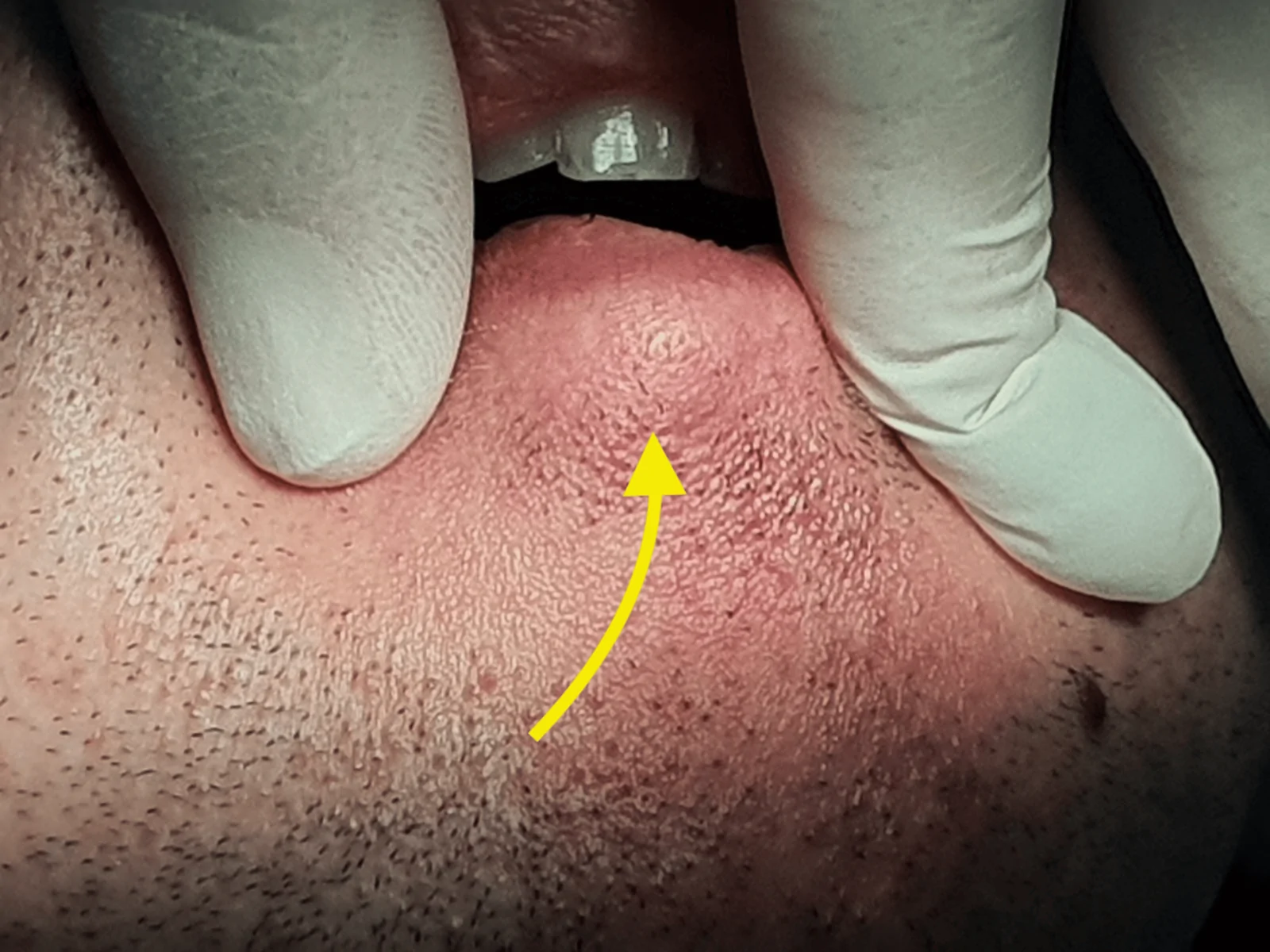
Clinical appearance at initial presentation. Clinical photograph showing a nodular swelling of the lower-lip mucosa (arrow).

Clinically, the lesion measured approximately 1 cm in diameter. The overlying mucosa was intact and appeared normal. There was no evidence of ulceration or surface change. On palpation, the mass was firm, mobile, and non-tender, with well-defined borders. No cervical lymphadenopathy was detected. Although the lesion had previously been considered a sebaceous cyst by his general dentist, the clinical findings were not fully consistent with this diagnosis. Other possibilities included a mucocele, an irritation fibroma, or a neoplasm arising from the minor salivary glands, including both benign and malignant tumors. In view of the persistence of the swelling and the uncertainty surrounding the diagnosis, complete surgical excision was undertaken for both diagnostic and therapeutic purposes. Given the small size, mobility, well-defined clinical borders, and accessibility of the lesion, preoperative imaging or needle biopsy was not considered necessary, as complete excision would allow definitive histopathological diagnosis while also serving a therapeutic purpose. The procedure was performed under local anesthesia using an intraoral approach. Following a mucosal incision over the lesion, a well-circumscribed nodular mass was identified within the submucosal tissue (Figure 2).

**Figure 2:**
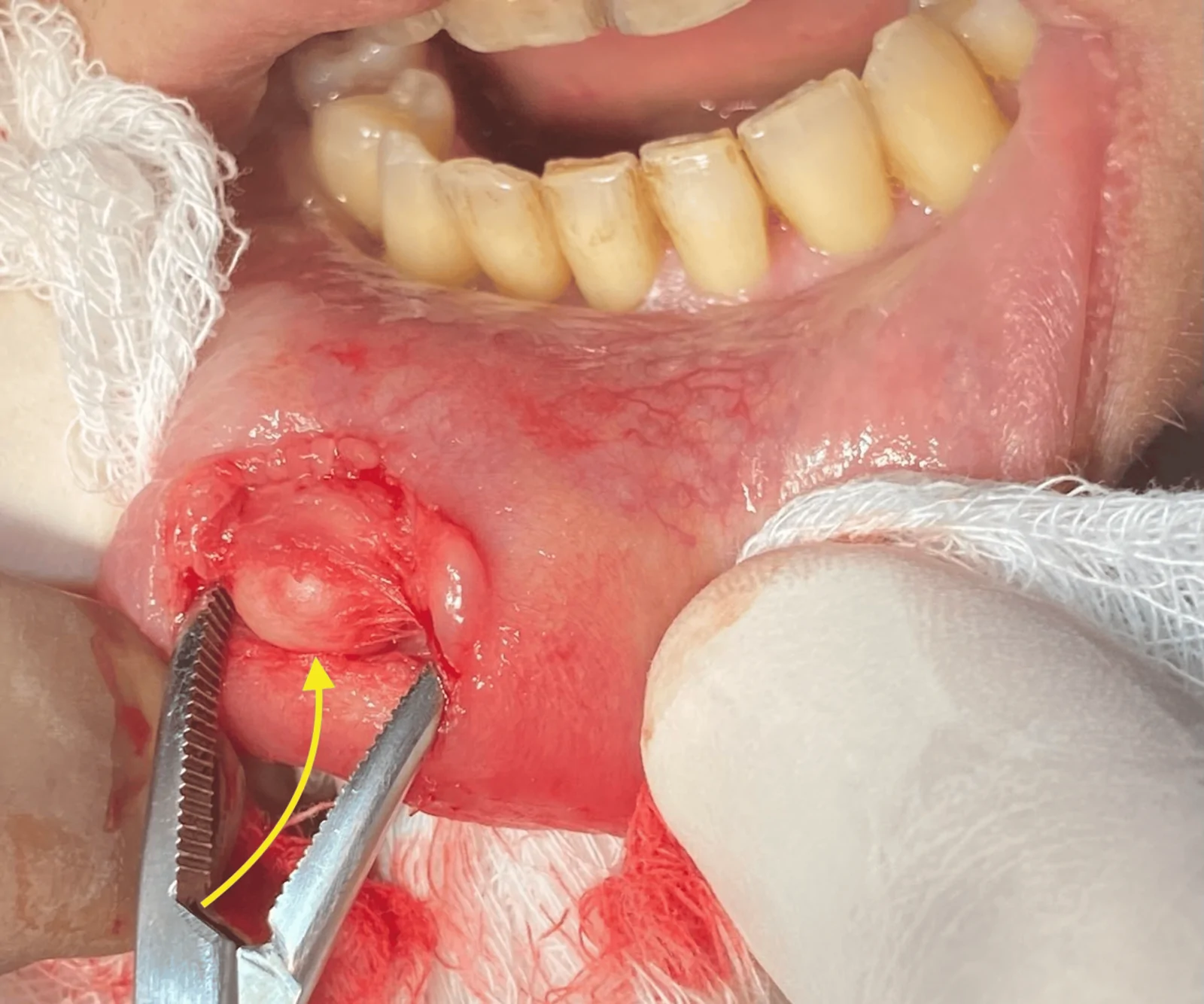
Intraoperative view of the lesion. Intraoperative photograph showing a well-defined nodular mass (arrow) within the submucosal tissue of the lower lip during surgical excision.

The lesion appeared encapsulated and was carefully dissected from the surrounding tissues before being excised in toto. The surgical site was then irrigated and closed with interrupted sutures. The specimen was submitted for histopathological examination.

On gross examination, the excised lesion measured 8 mm in greatest dimension. Histopathological examination with hematoxylin and eosin (H&E) staining demonstrated a well-circumscribed lesion composed of epithelial and myoepithelial cells arranged in duct-like structures within a chondromyxoid stromal background. No cytological atypia or features of malignancy were identified. The lesion was surrounded by a fibrous connective tissue capsule with smooth borders and was completely excised. The epithelial and myoepithelial components were identified morphologically on H&E-stained sections; no immunohistochemical examination was performed. The histopathological features established the diagnosis of pleomorphic adenoma of minor salivary gland origin (Figures 3, 4, 5).

**Figure 3:**
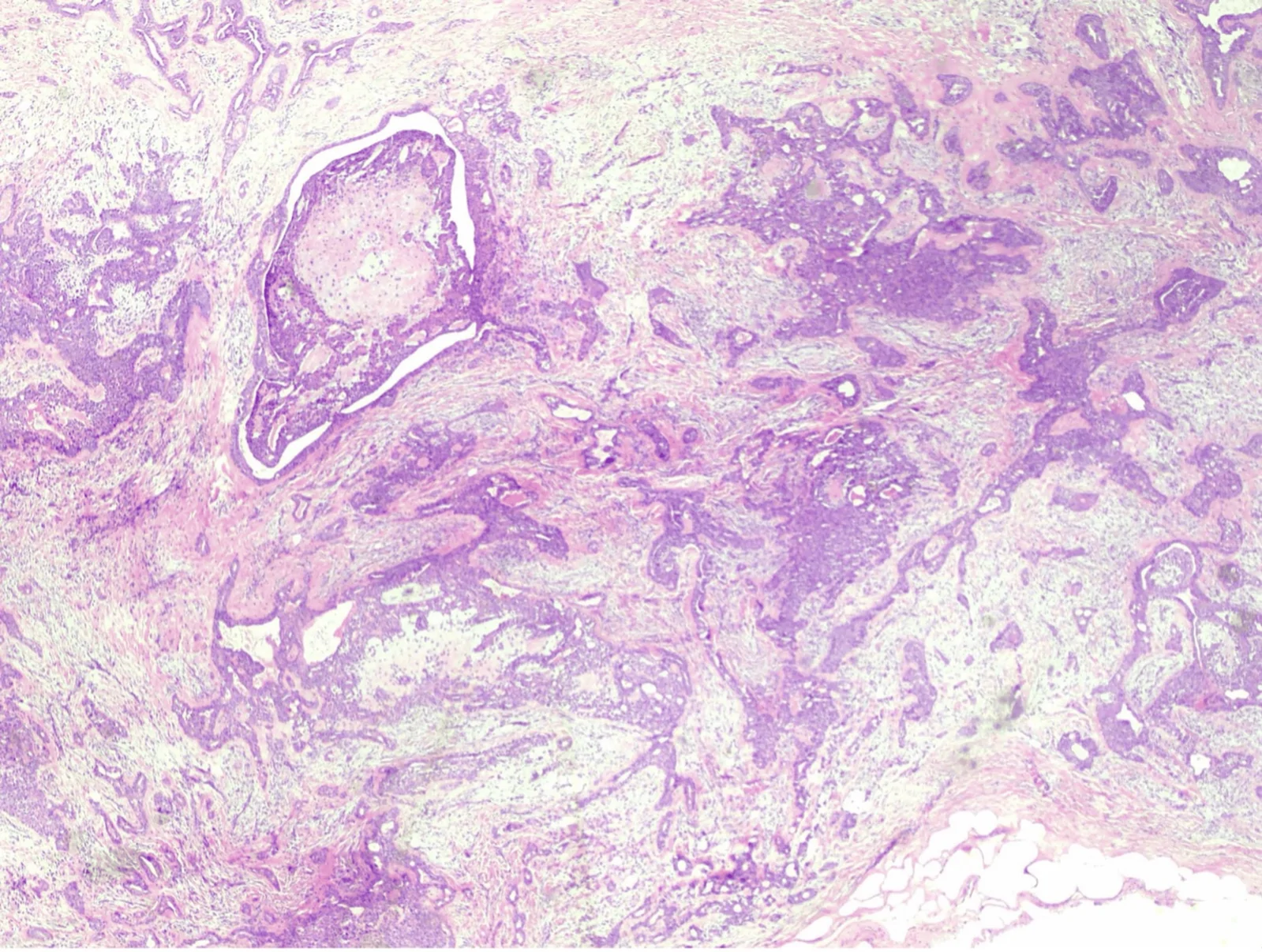
Histopathological features of pleomorphic adenoma (H&E, ×20). Photomicrograph showing the characteristic histopathological pattern of pleomorphic adenoma, comprising ductal (epithelial), myoepithelial, and stromal components, with prominent myxoid stroma.

**Figure 4:**
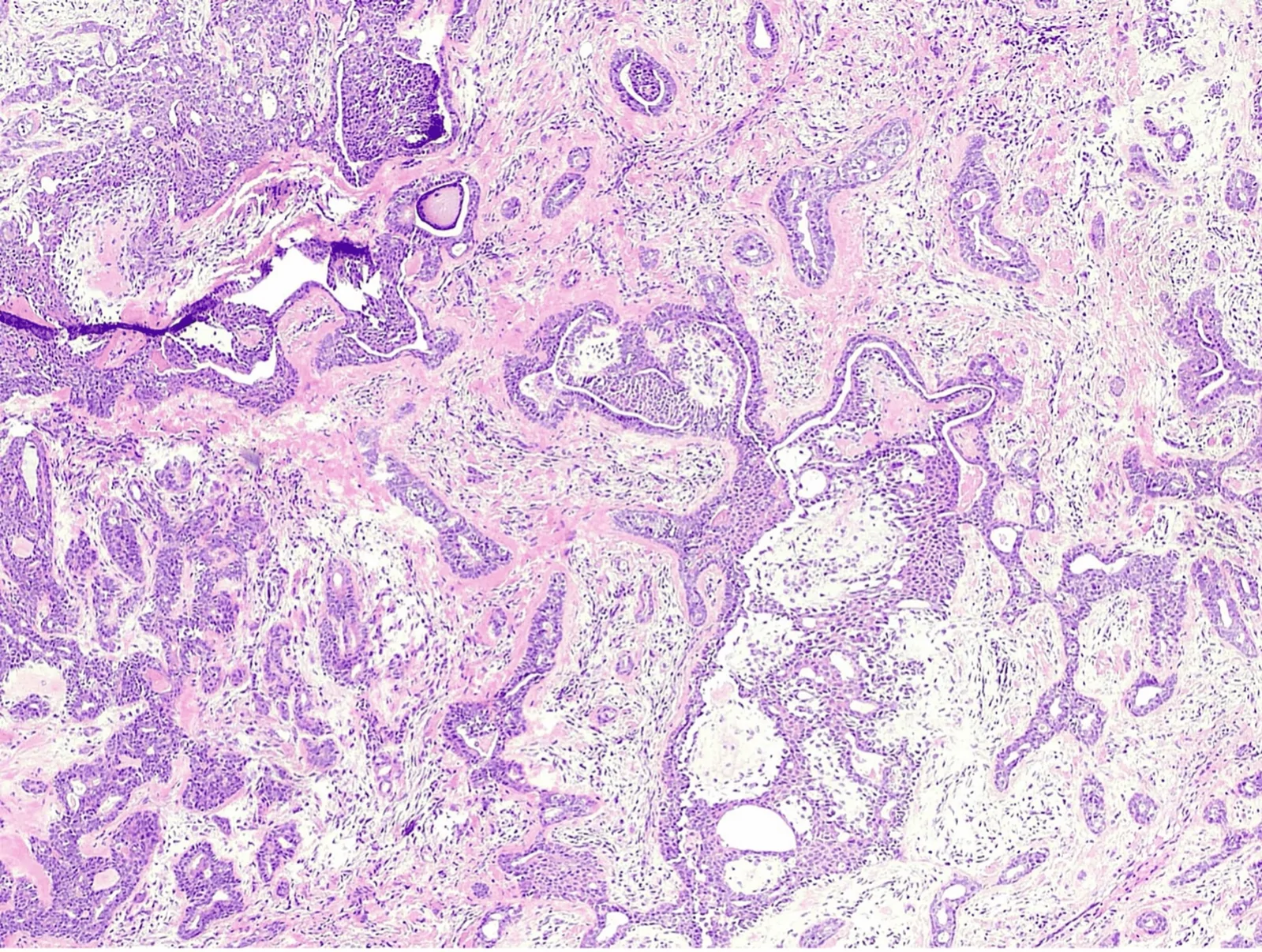
Histopathological features of pleomorphic adenoma (H&E, ×40). Higher-magnification photomicrograph showing duct-like epithelial structures surrounded by myoepithelial cells within a myxoid stromal background.

**Figure 5:**
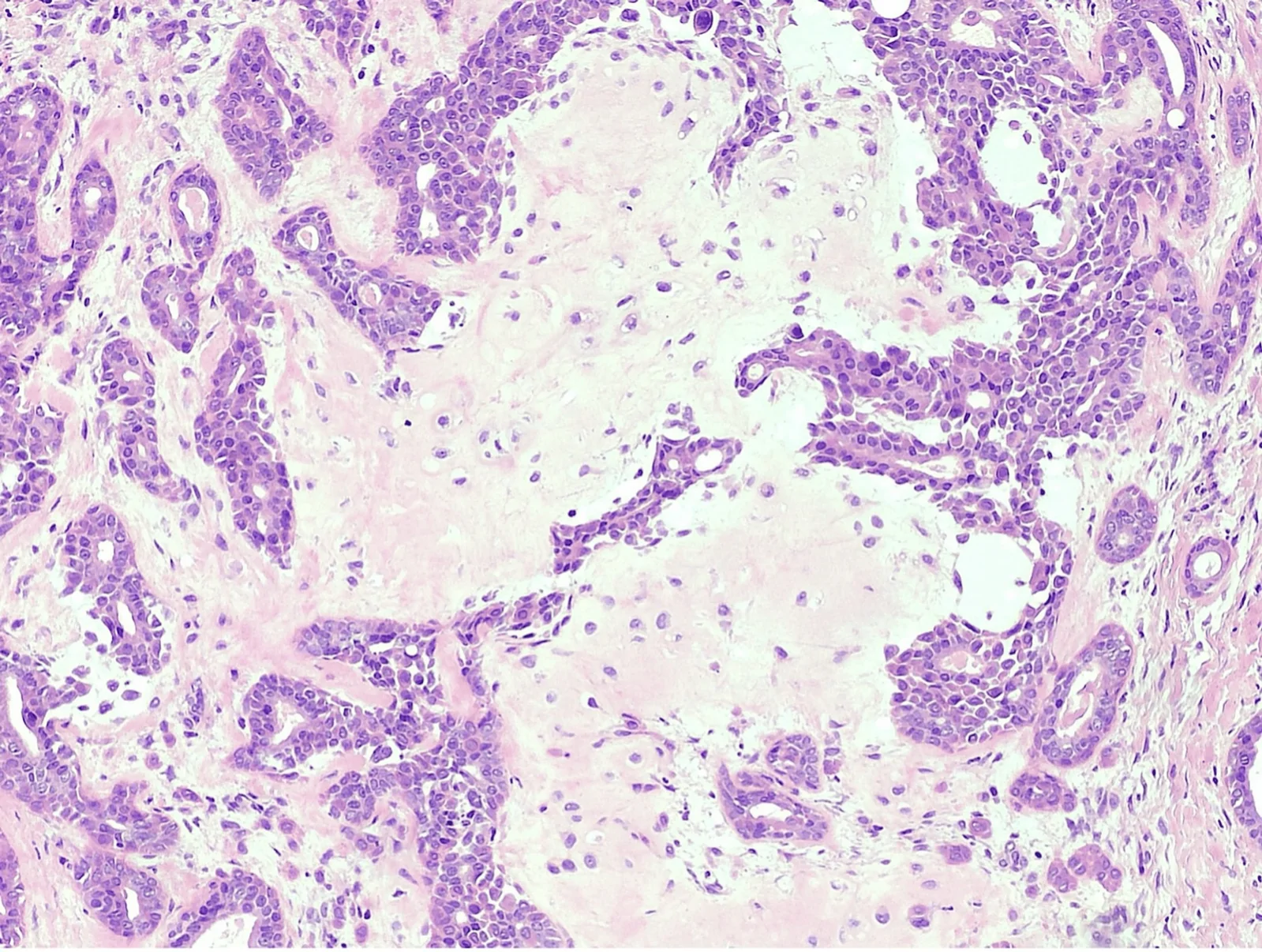
Histopathological features of pleomorphic adenoma (H&E, ×100). Higher-magnification photomicrograph showing ductal structures lined by inner epithelial cells and surrounded by an outer myoepithelial cell layer, embedded within a chondroid stromal background.

The postoperative course was uneventful. At the one-week follow-up, satisfactory healing of the surgical site was observed, with no signs of infection, inflammation, or wound dehiscence (Figure 6).

**Figure 6:**
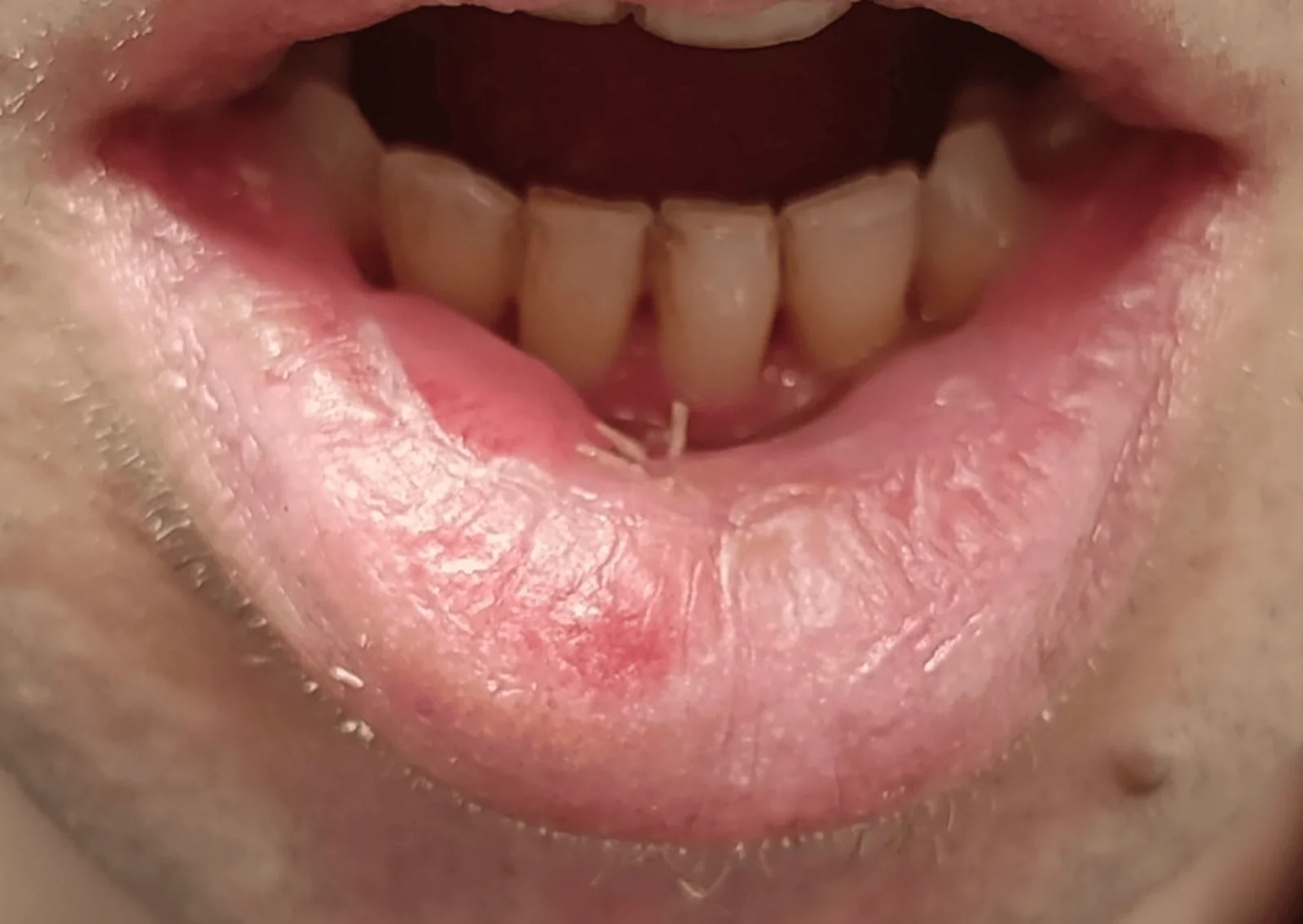
Postoperative appearance at one-week follow-up. Clinical photograph obtained one week after surgical excision showing satisfactory healing of the lower lip with no clinical signs of infection or wound dehiscence.

The patient was advised to undergo periodic clinical follow-up due to the risk of recurrence. At the three- and six-month follow-up visits, the patient remained asymptomatic, with no clinical evidence of recurrence. Annual clinical follow-up was planned thereafter.

## Discussion

Pleomorphic adenoma is the most common benign tumor of the salivary glands. It most often arises in the parotid gland, while a smaller proportion develops in the minor salivary glands of the oral cavity. A recent population-based study reported an incidence of around 4.79 cases per 100,000 person-years [4], with most cases occurring in middle adulthood. Clinically, pleomorphic adenoma usually presents as a slow-growing, painless mass and shows a slight female predominance [4]. On histological examination, the tumor consists of a mixture of epithelial and myoepithelial cells arranged in duct-like structures within a variable stromal background, which may exhibit myxoid, chondroid, or fibrous differentiation [5].

Pleomorphic adenomas arising from the minor salivary glands occur less frequently, with the palate being the most common intraoral site [6]. Other intraoral sites include the buccal mucosa, tongue, and lips, although these locations are less commonly involved [1]. Within the lips, pleomorphic adenomas show a marked predilection for the upper lip, whereas lower-lip involvement is uncommon [1,6-8], accounting for fewer than 3% of all intraoral pleomorphic adenomas [9]. As a result, lesions in this region may not always be suspected initially and can clinically mimic more common non-neoplastic lesions of the lower lip, such as mucoceles and irritation fibromas [9-11].

Pleomorphic adenoma typically presents as a slow-growing, painless, well-circumscribed submucosal mass, with the overlying mucosa usually remaining intact [9]. Such lesions are often firm and mobile on palpation and can remain asymptomatic for long periods before patients seek medical advice [1]. In lip lesions, pleomorphic adenomas are often relatively small and may present with nonspecific clinical features, making preoperative diagnosis more difficult [6,10,11]. In the lower lip, the nonspecific clinical appearance may complicate the preoperative diagnosis, and the differential diagnosis includes common benign lesions such as mucoceles and fibromas, benign salivary gland neoplasms such as canalicular adenoma, and malignant minor salivary gland tumors, including mucoepidermoid carcinoma and polymorphous adenocarcinoma [1,9-12]. In the present case, the referring general dentist initially considered the lesion a sebaceous cyst; however, its persistence, firm consistency, and submucosal location underscored the need to consider a minor salivary gland neoplasm despite its clinically benign appearance. Moreover, the empirical use of a topical corticosteroid, which is not an indicated treatment for a sebaceous cyst, further illustrates the potential consequences of an inaccurate initial clinical diagnosis and the importance of reassessing persistent lower lip lesions that do not respond to initial management.

Complete surgical excision is the standard treatment for pleomorphic adenomas arising in both major and minor salivary glands, with the aim of removing the tumor with adequate margins while preserving surrounding structures; capsular rupture, tumor spillage, or incomplete excision during surgery may increase the risk of recurrence [13]. Pleomorphic adenomas may exhibit microscopic pseudopod-like extensions beyond the tumor capsule, which may contribute to local recurrence when the lesion is not adequately removed [9]. Recurrence may occur many years after the initial treatment, and recurrent lesions are often more difficult to manage surgically, emphasizing the importance of long-term clinical follow-up [2,9]. Although pleomorphic adenoma is considered a benign neoplasm, long-standing or recurrent lesions may rarely undergo malignant transformation into carcinoma ex pleomorphic adenoma, a carcinoma arising from a pre-existing pleomorphic adenoma [3]. Therefore, complete excision and appropriate postoperative follow-up are essential to minimize the risk of recurrence and allow early recognition of potential complications. In the present case, no clinical evidence of recurrence was observed at the three- and six-month follow-up visits. However, this represents only short-term follow-up and does not exclude late recurrence; therefore, annual clinical surveillance was planned thereafter.

## Conclusions

Pleomorphic adenoma should be included among the possible diagnoses of a persistent submucosal lower lip lesion, even though it is uncommon at this site. Its nonspecific clinical presentation may lead to initial misdiagnosis, underscoring the importance of avoiding empirical treatment of persistent, undiagnosed lesions and obtaining histopathological examination for definitive diagnosis. The differential diagnosis should include both benign and malignant minor salivary gland neoplasms, as low-grade malignancies may present with clinically innocuous features. Complete excision is the treatment of choice. Although this case showed no clinical evidence of recurrence during the six-month follow-up period, this short-term observation does not exclude late recurrence, and long-term clinical surveillance is warranted.
